# Expression of DLX6 Gene in Mandibular Deficiency (Retrognathic Mandible): A Randomized Clinical and Genetic Study

**DOI:** 10.7759/cureus.13572

**Published:** 2021-02-26

**Authors:** Rajalakshmi S J, Nausheer Ahmed, Shashikala Kumari, Venkanna gudda Sreenivas Prasad, Lohit N Naik, Vinod Kumar

**Affiliations:** 1 Orthodontics and Dentofaical Orthopedics, Government Dental College and Research Institute, Bangalore, IND; 2 Orthodontics and Dentofacial Orthopedics, Government Dental College and Research Institute, Bangalore, IND; 3 Oral and Maxillofacial Surgery, Yadgir Government District Hospital, Yadgir, IND; 4 Pedodontics, Navodaya Dental College and Hospital, Raichur, IND

**Keywords:** dlx6 gene, genetic evaluation, retrognathic mandible

## Abstract

Introduction

There are various genes that affect craniofacial development and among the important genes that affect jaw development is distal-less homeobox (DLX) 6 genes. The present study was carried out to determine the role of DLX6 gene variations in mandibular deficiency.

Methods

Thirty subjects having retrognathic mandible were evaluated by clinical examination and assessed using lateral cephalometric radiographs based on cephalometrics for orthognathic surgery (COGS) analysis of hard tissue with N-Pog parameters being less than -13 mm. For the same subjects, saliva samples were taken and sent to biotechnology labs for genetic evaluation. DNA was isolated from salivary samples using a DNA extraction kit and was subjected to polymerase chain reaction (PCR) amplification and sequencing. Single nucleotide polymorphisms (SNP) analysis was done to assess the role of DLX6 gene in these study subjects.

Results

All 30 subjects showed N-POG parameters of COGS analysis for hard tissue to be less than -13mm, confirming retrognathic mandible. SNP analysis of subjects showed no SNPs in any EXON of the DLX6 gene for all 30 study samples.

Conclusion

No variations in DLX6 gene were found in the present study. Further studies are required to investigate other genes that could be involved in the cause of retrognathic mandible with a larger sample size and to include subjects in the sample having features other than mandibular retrognathia like hearing loss, abnormal pinnae, ectrodactyly, cleft palate, developmental delay and abnormal teeth to determine the contribution of DLX6 gene variations in mandibular deficiency.

## Introduction

There are various genes that affect craniofacial development and among the important genes that affect jaw development is distal-less homeobox (DLX) 6 genes. DLX genes comprise a highly conserved family of homeobox genes homologous to that distal-less (DLL) gene of drosophila. They are thought to act as transcription factors. All DLX genes are expressed in spatially and temporally restricted patterns in craniofacial primordia, basal telencephalon and diencephalon and in distal regions of extending appendages, including the limb and the genital bud. DLX 5 and DLX 6 are expressed in differentiating osteoblasts [1]. Studies have shown that DLX 5 and DLX6 were expressed in all skeletal elements from initial cartilage formation until ossification in mice [2]. In gnathostome jaws, DLX5 and DLX6 expression in neural crest cells of pharyngeal arch 1 invariably determines lower jaw identity and maintains the myogenic program for the formation of muscularized jaws [3]. In humans, three generations of the family showed five affected members all of whom had hearing loss, micrognathia, abnormal pinnae and paracentric inversion of the long arm of chromosome 7. Deregulation of DLX5 and DLX 6 genes were implicated in the pathogenesis of the family’s phenotype [4].

The literature on the role of DLX6 gene as a single entity resulting in mandibular deficiency is insufficient. This study will be helpful for the orthodontists to formulate treatment plans for mandibular deficiency-associated malocclusion and it is also useful for genetic scientists and orthopedicians as a source of complementary information. Hence this study centralized on evaluating DLX6 gene variations contributing to mandibular deficiency.

## Materials and methods

**Figure 1:**
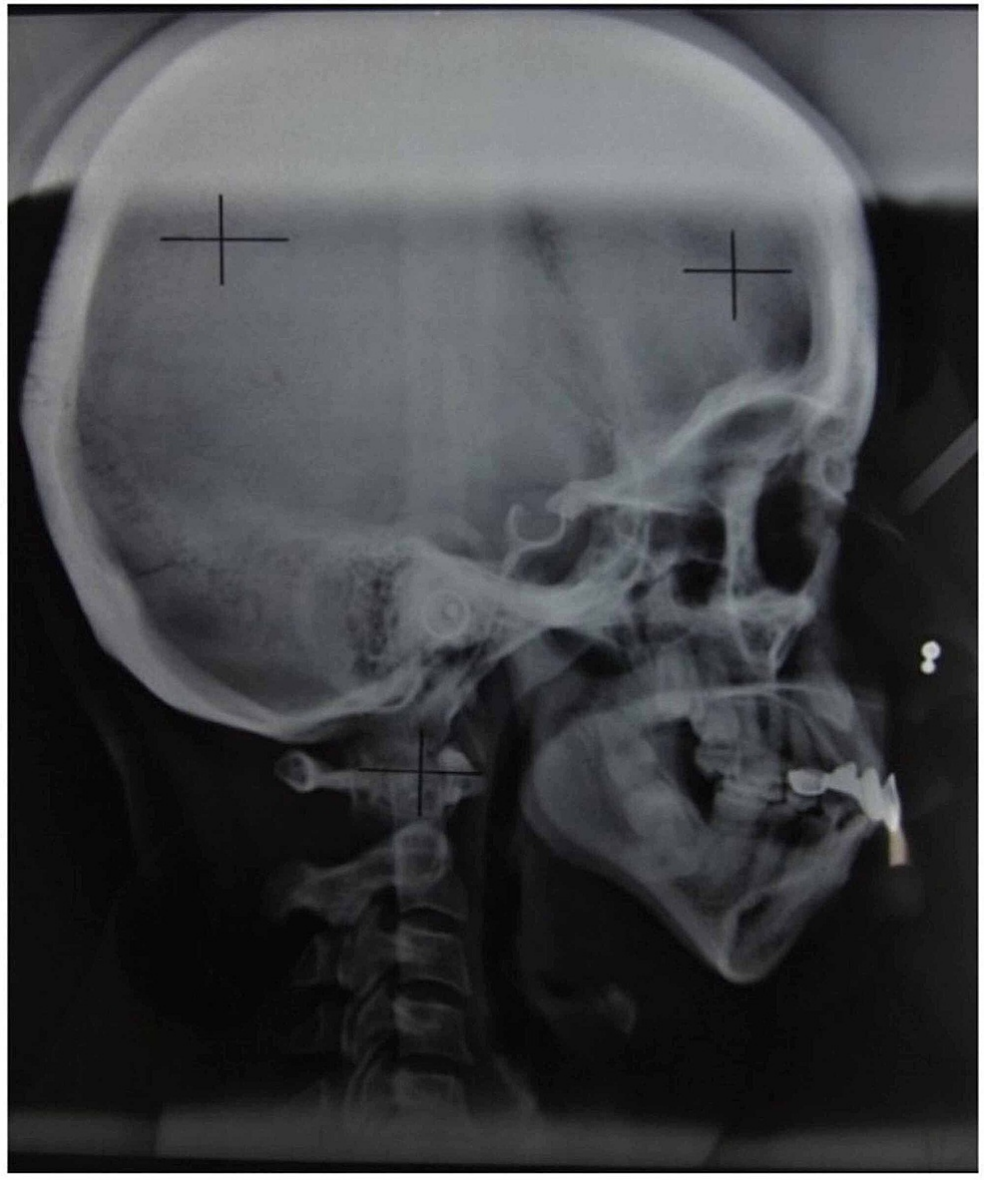
Lateral cephalogram.

**Figure 2:**
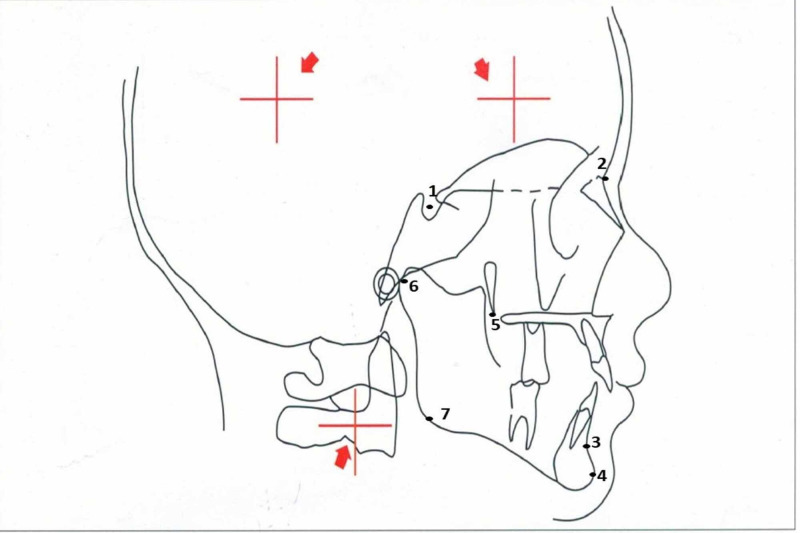
Cephalometric landmarks used in this study.

It’s a pilot study. Thirty study subjects with retrognathic mandible (confirmed by cephalometric radiographs) aged between 18 and 60 years were selected by a simple random sampling method. The syndrome patients, systemic diseases patients, and uncooperative patients were excluded. Rights of human subjects were protected; informed consent obtained from the patients and this study was approved by the institutional ethical committee of Government Dental College and Research Institute, Bangalore, India. This study was conducted from December 2010 to November 2012. Detailed case histories were elicited from the study subjects. Subjects with retrognathic mandible were evaluated by clinical examination and confirmed using lateral cephalometric radiographs based on cephalometrics for orthognathic surgery (COGS) analysis of hard tissue with N-Pog parameters being less than -13 mm. Figure 1 shows the lateral cephalogram of a female patient. Figure 2 shows cephalometric landmarks used in this study.

Landmarks used as shown in Figure 2: Sella (S) - The center of Pituitary Fossa, Nasion (N) - The most anterior point of the nasofrontal suture in the mid-sagittal plane, supramentale (B) - The deepest point in mid-sagittal plane on the concavity between infradentale and pogonion, Pogonion (Pg) - Most anterior mid-sagittal point on the contour of the chin, Pterygomaxillary fissure (Ptm) - The most posterior point on the anterior contour of the maxillary tuberosity, Articulare (Ar) - The intersection of basisphenoid and the posterior border of the condyle, Gonion (Go) - Constructed by bisecting the posterior ramal plane and mandibular plane.

For the same 30 subjects, unstimulated saliva samples were collected and stored in a refrigerator at -20 degree centigrade. 5-ml normal saline was given to the patient to hold it in mouth for one minute and then spit it back to sterile sample collection falcon tube. Samples were sent to the biotechnology laboratory within seven days (Chromous Biotech Pvt. Ltd., Bangalore). DNA was isolated from salivary samples using a DNA extraction kit and was subjected to PCR (polymerase chain reaction) amplification. Once the gene was amplified, it was subjected to DNA sequencing to check for single nucleotide polymorphisms (SNP). It was done to see the role of DLX6 gene in these cases. DLX6 gene is a homeobox gene located on chromosome 7q22. It has 5,062 base pairs which include exons and introns. Exons are the protein-coding regions of the gene. Introns are the non-coding regions of the gene. DLX6 gene has 3 exons. 1st exon has 82 base pairs, 2nd exon has 200 base pairs and 3rd exon has 255 base pairs.

Procedure followed by the biotechnology lab for the gene evaluation:

Genomic DNA (gDNA) was isolated from the samples provided by using a DNA extraction kit. 2 µl of the Genomic DNA isolated was loaded on 1 % Agarose gel.

PCR amplification of the gene was performed. Primers were designed by using the reference sequence of the DLX6 gene of the human for all the 3 exons of DLX6 gene.

The PCR Mix of total 50µl was prepared which had 1µl template DNA, Forward Primer 2µl (200ng), Reverse Primer 2µl (200ng), dNTPs (2.5mM each) 2 µl, 10X Taq DNA polymerase Assay Buffer 5 µl, Taq DNA Polymerase Enzyme (3U/µl)-0.5µl, and Water 37.5µl.

The Exons 1, 2 and 3 of DLX6 gene was amplified by 35 cycles consisting of denaturation at 94°C for 30 sec, with an initial denaturation for 5 min, annealing at 55°C for 30 sec, and primer extension at 72°C for 1 min, with final extension for 5 minutes.

Reaction was cycled in a thermal cycler.

**Figure 3:**
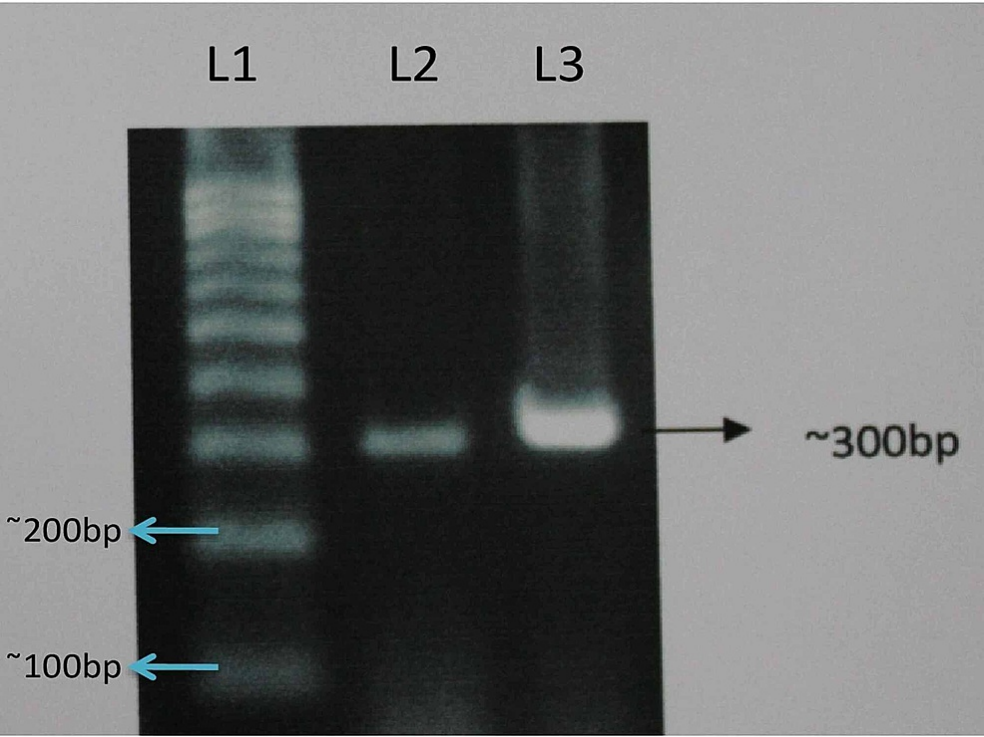
Post-PCR products on 1% agarose gel. Grey bands represent the wells the product (gDNA isolated from the saliva sample of the subject) was loaded into, and the white bands represent DNA fragments produced by PCR.  In this 100bp DNA ladders (Lanes 1, 2 and 3), the target fragments amplified by the primers were 300bp in size. Lane 1 represents DNA ladder marker; Lanes 2 and 3 represent PCR amplified products of DLX6 gene of the subject.

PCR products were then loaded on 1% Agarose gel and post-PCR products have been shown in Figure 3. Sequencing of the PCR product was done by using ABI Sequencing machine ABI 3500 XL Genetic Analyzer.

The demographic details of 30 study participants like age, gender and COGS were subjected to descriptive statistics (percentage, mean, range, and standard deviation).

## Results

The following were the results of this study: Out of 30 subjects, 15 were males and 15 were females.

For females, the age ranged from 18 years to 42 years with the mean age being 24 years and standard deviation was 10.20 years. For males, the age ranged from 18years to 59 years with the mean age being 29.2 years and standard deviation was 14.62 years.

For females, the N-Pog parameter of COGS analysis ranged from -16 mm to -23 mm with the mean being -20.40 mm and standard deviation was 3.21 mm. For males, the N-POG parameter of COGS analysis ranged from -17 mm to -22 mm with the mean being -19.36 mm and standard deviation was 1.75 mm as shown in Table 1.

**Table 1:** Mean N-pog parameter of study sample.

Gender	N-pog parameter in range (mm)	Mean ± SD (mm)
Female	-16 to -23	-20.40 ± 3.21
Male	-17 to -22	-19.36 ± 1.75

**Figure 4:**
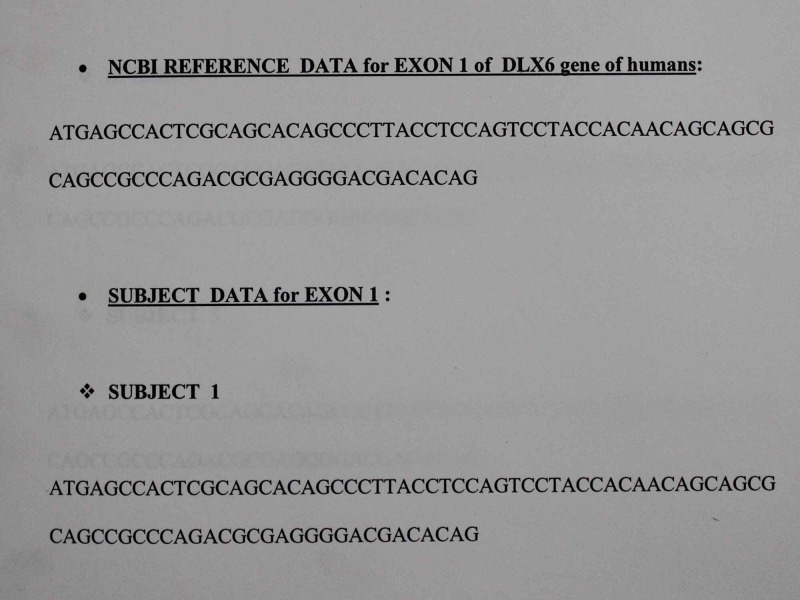
NCBI reference data for exon 1 of DLX6 gene in humans.

Genetic evaluation results: SNP analysis was done by comparing the reference data from NCBI (NCBI reference sequence number for DLX6 gene on chromosome 7: NC_000007.13) with the sequence data obtained from the 30 samples for all the three exons (exon 1, 2, 3) of DLX6 gene by using the Align tool of Blast in NCBI and analyzing the mismatches if any to detect SNP (Figure 4). It showed no SNPs in any of the exons of the DLX6 gene of all 30 samples indicating non-involvement of DLX6 gene in mandibular deficiency.

## Discussion

Studies have shown that DLX5 and DLX6 are predominantly expressed by mandibular cranial neural crest cells, which give rise to most bones and tendons of jaws [5]. It is documented in chick embryos that levels of DLX5 and DLX6 are required for the maintenance of cellular proliferation during development and over expression of DLX5 and DLX6 leads to generalized antagonism of cellular proliferation [6]. Evaluation of phenotype of DLX5 and DLX6 double mutant mice suggest they specify mandibular identity [5]. A study on gnathostome jaws shows that neural crest cells pharyngeal arch 1 specific DLX5 and DLX6 inactivation generates severely hypomorphic lower jaws that have typical maxillary traits [3]. The role of DLX5 and DLX6 was evaluated in mice. The inactivation of DLX5 and DLX6 in mice had shown a multitude of craniofacial and ear defects including the failure of Meckel’s cartilage, mandible, calvaria formation [7]. A study on dogs has shown that a LINE-1 insertion within DLX6 is responsible for cleft palate and mandibular abnormalities, which prompted sequencing of DLX5 and DLX6 in humans with Pierre Robin Sequence, where a missense mutation within the highly conserved DLX5 homeobox was identified [8]. A study on humans has shown multiple individuals with deletion of DLX5 and DLX6 who had reported severe phenotypes including ectrodactyly, hearing loss, abnormal pinnae, cleft palate, lower jaw retrognathia, developmental delay and abnormal teeth [9]. So DLX6 gene is an important developmental gene responsible for proper mandibular jaw development in humans and its variations may lead to failure of proper development of mandibles in them. In the present study, since no SNPs were found in DLX6 gene of the study subjects, the causative factor is other than the SNPs, as retrognathic or a small mandible has a multifactorial etiology. Human study done shows DLX6 gene affected members to be having features other than mandibular retrognathia like hearing loss, abnormal pinnae, ectrodactyly, cleft palate, developmental delay and abnormal teeth. The samples with any one of the above features if subjected to genetic evaluation probably would show mutations in DLX6 gene.

Variation of the phenotype is a central issue in biology because it is the basis for individuality, adaptation of populations to environmental fluctuations, and the evolution of biodiversity. Phenotypic variation can be produced by genetic differences, environmental influences and stochastic developmental events [10]. The concept that phenotype represents the consequence of genotype-environment interactions (GEI) is universal and relates to all living organisms. In response to severe environmental changes, a genome can respond by selectively regulating (increasing or decreasing) the expression of specific genes [11]. So in the present study even though DLX6 gene variation or SNPs is not present, other genes or environmental factors could have influenced and resulted in retrognathic mandible in these subjects.

## Conclusions

It was concluded in the present study that DLX6 gene was not the cause for retrognathic mandible in the subjects of the present study. Retrognathic mandible has a multifactorial etiology so further studies are required to investigate other genes that could be involved in the cause of retrognathic mandible and to include subjects with different ethnicity in the sample who are also having features other than mandibular retrognathia like hearing loss, abnormal pinnae, ectrodactyly, cleft palate, developmental delay and abnormal teeth to determine the contribution of DLX6 gene variations in mandibular deficiency.
